# Effects of bilirubin on the development and electrical activity of neural circuits

**DOI:** 10.3389/fncel.2023.1136250

**Published:** 2023-03-21

**Authors:** Cuiping Wu, Yuefan Jin, Yaqi Cui, Yidan Zhu, Shankai Yin, Chunyan Li

**Affiliations:** Shanghai Key Laboratory of Sleep Disordered Breathing, Department of Otolaryngology – Head and Neck Surgery, Otolaryngology Institute of Shanghai Jiao Tong University, Shanghai Sixth People’s Hospital Affiliated to Shanghai Jiao Tong University School of Medicine, Shanghai, China

**Keywords:** bilirubin, neural circuits, electrical activity, ion channel, synaptic transmission, excitotoxicity, neurological dysfunction

## Abstract

In the past several decades, bilirubin has attracted great attention for central nervous system (CNS) toxicity in some pathological conditions with severely elevated bilirubin levels. CNS function relies on the structural and functional integrity of neural circuits, which are large and complex electrochemical networks. Neural circuits develop from the proliferation and differentiation of neural stem cells, followed by dendritic and axonal arborization, myelination, and synapse formation. The circuits are immature, but robustly developing, during the neonatal period. It is at the same time that physiological or pathological jaundice occurs. The present review comprehensively discusses the effects of bilirubin on the development and electrical activity of neural circuits to provide a systematic understanding of the underlying mechanisms of bilirubin-induced acute neurotoxicity and chronic neurodevelopmental disorders.

## 1. Introduction

Neonatal jaundice affects 60–80% of newborns and is mainly due to overproduction of bilirubin and immaturity of hepatic enzymes for bilirubin clearance, which is usually physiological and transitional (Fujiwara et al., 2018; Olusanya et al., 2018). However, in cases with extremely high serum bilirubin concentrations or other risk factors (Olusanya et al., 2018), such as prematurity, sepsis, acidosis, and hypoxia, unconjugated bilirubin (UCB) can accumulate in the brain, particularly in the globus pallidus, subthalamic nucleus, brainstem nuclei, hippocampus, and cerebellum. This causes acute or chronic bilirubin encephalopathy, also known as kernicterus, which can lead to motor and auditory disorders, and even death. Several studies have demonstrated that hyperbilirubinemia is associated with long-term neurodevelopmental disorders, including auditory spectrum disorders (Shapiro and Popelka, 2011; Amin et al., 2017), cerebral palsy (Wu et al., 2015), cognitive abnormalities (Mwaniki et al., 2012; Hokkanen et al., 2014), autism spectrum disorders (ASDs) (Maimburg et al., 2010), and attention deficit hyperactivity disorder (ADHD) (Jangaard et al., 2008).

Brain function is mediated by neural circuits consisting of neurons connected by synapses (Purves et al., 2012). Neural circuits emerge in the embryonic period and continue to develop rapidly in the perinatal period (Tau and Peterson, 2010). The network relies on neuronal electrical activity to transmit and process information. Neural circuits are particularly vulnerable to bilirubin toxicity during the perinatal period because developmental processes occur within narrow time windows. Bilirubin-induced developmental defects and neural circuit dysfunction are thought to be associated with long-term neurodevelopmental disorders (Koziol et al., 2013; Amin et al., 2019; Hansen et al., 2020). The present study reviews the current state of knowledge regarding the mechanisms of bilirubin-induced neural circuit damage, with a particular focus on neurodevelopment and electrical activity, and discusses the potential future directions for bilirubin research.

## 2. Bilirubin and neural circuit development

**FIGURE 1:**
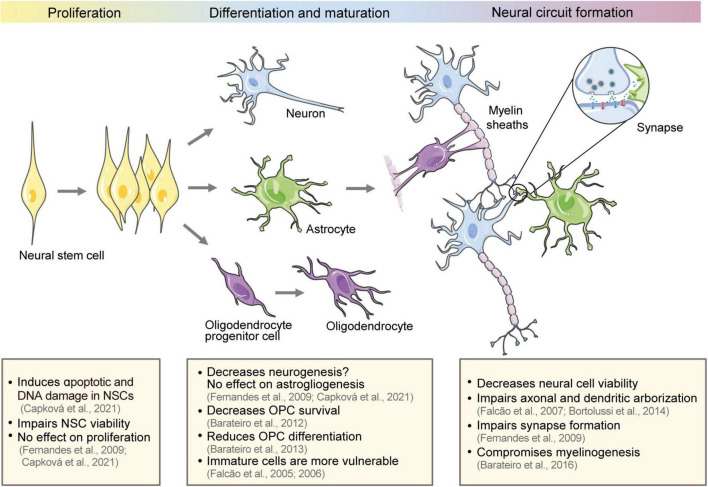
Effect of bilirubin on the development of neural circuits. The schematic diagram shows the development of neural circuits and the effect of bilirubin on different development stages, including NSC proliferation and differentiation, neural cell maturation, and neural circuit formation. NSCs, neural stem cells; OPCs, oligodendrocyte progenitor cells.

Neural circuits originate from neural stem cells (NSCs) in the embryonic period. NSCs proliferate and differentiate into different neural cell lines that undergo synaptogenesis, myelination, and refinement to form neural circuits (Figure 1). Neural circuit alterations during development trigger a cascade of negative effects and cause neurodevelopmental disorders (Del Pino et al., 2018).

### 2.1. Effect of bilirubin on NSC proliferation and differentiation

Neural stem cells are ectodermal, uncommitted progenitor cells with the capacity for self-renewal to enlarge the stem cell pool. They are multipotent cells that can generate neurons, astrocytes, oligodendrocytes, and committed progenitors (Kriegstein and Alvarez-Buylla, 2009). NSC proliferation and differentiation continues into the third trimester of pregnancy and, in certain regions, postnatally (Malik et al., 2013; Vieira et al., 2018). Previous studies have reported that the exposure of embryonic stem cell-derived NSCs to UCB impairs their metabolic activity, increases apoptotic and DNA-damage-related molecular markers, and decreases NSC viability (Fernandes et al., 2009; Capková et al., 2021). Meanwhile, the effect on NSC proliferation seems to be minimal. Capková et al. (2021) reported that 12.5 μM of bilirubin did not affect the cell cycle of NSCs. However, bilirubin can induce cell cycle alterations in other cell types, including neural and non-neural cells, as reported in previous studies (Ollinger et al., 2005, 2007; Deganuto et al., 2010; Robert et al., 2013). There is contradictory evidence as to whether bilirubin affects NSC differentiation. Fernandes et al. (2009) reported that UCB decreased the differentiation into neurons by 52.8%, but did not affect the differentiation into astrocytes. Meanwhile, Capková et al. (2021) reported that bilirubin did not cause any alterations in the terminal differentiation into neurons or astrocytes. The differences between these studies may be related to the concentration of bilirubin and the duration of exposure. Therefore, the exact effects of bilirubin on NSCs remain unknown. Complex models, such as brain organoid models and *in vivo* studies, may efficiently mimic the complex regulatory environment for NSC fate determination and may be used to determine the effects of bilirubin on NSC development (Pranty et al., 2022).

### 2.2. Effect of bilirubin on neuron maturation

Neuron maturation follows neurogenesis and continues during the third trimester of gestation in humans and the first 10 days after birth in rodents. Maturation includes extending and elaborating axons and dendrites, forming and pruning back synapses, and eventually establishing precise neural networks (Salmaso et al., 2014). Several studies have demonstrated that immature neurons and glial cells are more sensitive to UCB compared to mature cells (Falcao et al., 2005, 2006). The susceptibility of immature cells partially explains the tendency of preterm infants to develop kernicterus at bilirubin levels that are generally considered nonhazardous (Watchko, 2016).

Bortolussi et al. (2014) also suggested that the degree of bilirubin-induced impairment depends on the developmental stage of cells. They induced null mutations of *Ugt1*, which encodes UDP-glucuronosyltransferase 1a1, an enzyme that transforms bilirubin into water-soluble and excretable metabolites, in FVB/NJ mouse strains to generate a neonatal hyperbilirubinemia model. Central nervous system (CNS) sensitivity to bilirubin was investigated by withholding phototherapy in *Ugt1^–/–^* mice at different neonatal periods to control bilirubin exposure. They concluded that the window for greatest toxicity was postnatal day 0–8 (P0–P8). Bilirubin toxicity during P0–P8 caused apoptosis of cerebellar granule and Purkinje neurons, reduced the thickness of the external germinal layer (EGL) and Purkinje cell (PC) layer in the cerebellum and destroyed PC dendrites. Soon after birth in rodents, granule neuron precursors proliferate vigorously in the EGL and subsequently migrate radially to internal granule layer (IGL) as mature granule cells. Granule cells extend axons into the molecular layer to form synapses with PC dendrites (Butts et al., 2014). Bilirubin toxicity during this period may impair the generation and maturation of cerebellar neurons. Therefore, the changes induced by bilirubin during P0–P8 could not be rescued by subsequent phototherapy and further resulted in a reduction in the thickness of the molecular layer and IGL at P15 (Bortolussi et al., 2014). In humans, the cerebellum develops during the 24th–38th gestational weeks (Biran et al., 2012). Therefore, prompt treatment of hyperbilirubinemia is important in preterm infants.

*In vitro* studies have shown that bilirubin impairs neuron maturation, including cell viability, dendritic and axonal arborization, axonal growth cone morphology, dendritic spine formation, and synapse establishment (Falcao et al., 2007; Fernandes et al., 2009). Tau, a microtubule-associated structural protein, is released extracellularly in response to axonal injury (Magnoni et al., 2012). The neurites damage by bilirubin is consistent with the increase in serum tau levels when total serum bilirubin levels exceed 19.1 mg/dL in jaundiced newborns (Okumus et al., 2008). Generally, presynaptic terminals are formed on axons, while postsynaptic terminals are formed on dendritic spines (excitatory synapses) or dendritic shafts and neuronal soma (inhibitory synapses). The structural destructions of neurites caused by high bilirubin levels significantly reduces the formation of synapses (Fernandes et al., 2009). Silva et al. (2012) reported that the reduction in dendritic and axonal arborization may be prevented by MK-801 [an N-methyl-D-aspartate (NMDA) glutamate-subtype receptor antagonist] or L-NAME (a non-specific nitric oxide synthase inhibitor). This indicates that the impairment mechanism involves the activation of NMDA receptors and nitric oxide production. In particular, bile acid glycoursodeoxycholic acid (GUDCA) can prevent neuronal death, morphogenesis impairment, and reduction in the expression of the presynaptic proteins synaptophysin and SNAP-25 (Silva et al., 2012).

### 2.3. Effect of bilirubin on oligodendrocyte maturation

In addition to neurons, bilirubin also affects the differentiation and maturation of the oligodendrocyte lineage. In the mammalian CNS, oligodendrocyte progenitor cells (OPCs) proliferate and differentiate into mature oligodendrocytes, and finally form myelin sheaths that wrap and insulate the axons. This facilitates rapid saltatory conduction of action potentials and provides metabolic support for axons (Simons and Nave, 2015). White matter undergoes significant maturation during the last weeks of gestation and the first few postnatal months, making it extremely vulnerable to injury (Brites and Fernandes, 2015). Barateiro et al. (2012, 2013, 2014, 2016) conducted a series of experiments to explore the effects of bilirubin on oligodendrocyte lineage. They found that 50 μM of UCB with 100 μM of human serum albumin decreased OPC survival (Barateiro et al., 2012), prevented their transition to mature oligodendrocytes, impaired morphological maturation, and reduced myelinogenesis in primary OPC cultures (Barateiro et al., 2013). Hyperbilirubinemia also led to myelination deficits in the cerebellums of neonatal kernicterus mice (Barateiro et al., 2016). The AMPA receptor antagonist, 6-cyano-7-nitroquinoxaline-2,3-dione (CNQX), and TNF-α antibodies partially rescued bilirubin-induced myelination defects in organotypic cerebellar slice cultures (Barateiro et al., 2014). Myelin damage can slow network activity, interfere with neural conduction, and render the network inoperative. White matter abnormalities may affect cognitive proficiency and cause neuropsychiatric deficits (Cainelli et al., 2020). However, a cross-sectional study of 1,121 Japanese adults showed that low total bilirubin levels (<0.5 mg/dL) were associated with a high prevalence of severe deep white matter lesions (Higuchi et al., 2018). Low bilirubin levels were also found in multiple sclerosis patients (Peng et al., 2011; Ljubisavljevic et al., 2013), indicating that bilirubin may have a physiologically protective effect on white matter.

## 3. Bilirubin and synapses

**FIGURE 2:**
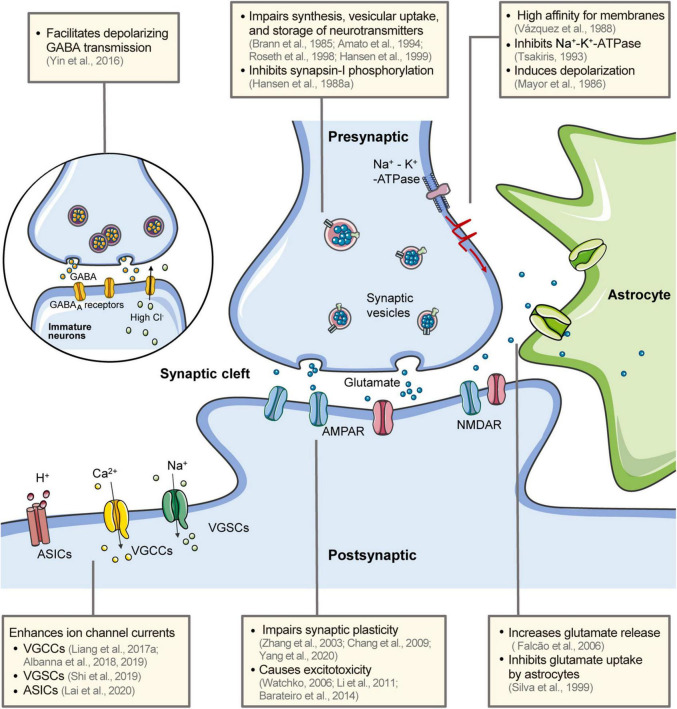
Effects of bilirubin on neural electrical activity. Bilirubin has a high affinity for the membrane and induces rapid depolarization and inhibits the Na^+^-K^+^-ATPase. It interferes with the synthesis, uptake, storage, and release of neurotransmitters in the presynaptic terminal. Glutamate release is increased and astrocyte uptake is inhibited. The excess glutamate causes excitotoxicity in postsynaptic neurons *via* glutamate receptors. Synapse plasticity is also impaired by bilirubin. In immature neurons, bilirubin facilitates transmission in GABAergic synapses and contributes to the hyperexcitability of postsynaptic neurons. Bilirubin can directly affect several ion channels in neurons, including VGCCs, VGSCs, and ASICs. Bilirubin has a high affinity for the membrane and induces rapid depolarization and inhibits the Na^+^ -K^+^ -ATPase (Mayor et al., 1986; Vazquez et al., 1988). GABA, gamma-aminobutyric acid; AMPAR, α-amino-3-hydroxy-5-methyl-4-isoxazolepropionic acid (AMPA) receptors; NMDAR, N-methyl-D-aspartate (NMDA) receptors; ASICs, acid-sensing ion channels; VGCCs, voltage-gated calcium channels; VGSCs, voltage-gated sodium channels.

Chemical synapses are the basis for inter-neuron communication. Output synapses convert spike codes into neurotransmitters in response to action potentials from presynaptic neurons (Sudhof, 2021). These chemical signals are transmitted to postsynaptic neurons, where they are again transformed into electrical signals and generate or inhibit action potentials in downstream neurons. The synapses are often classified according to vesicular transporters and enzymes in the presynaptic terminals or receptors in the postsynaptic terminals. They can also be broadly classified as excitatory or inhibitory based on their effect on postsynaptic neurons. The balance between excitatory and inhibitory synaptic transmission is important for information processing and the plasticity of neural circuits. Disturbances in this balance can cause neurodevelopmental and psychiatric disorders (Sohal and Rubenstein, 2019). Bilirubin interferes with the metabolism of various neurotransmitters and enhances excitatory synaptic transmission, leading to synaptic dysfunction and neural excitotoxicity (Figure 2).

### 3.1. Effect of bilirubin on synaptic transmission

The metabolism of neurotransmitters, including presynaptic uptake, synthesis, release, and clearance, is critical for orderly and effective synaptic transmission. Bilirubin has been reported to affect these metabolic processes. Neurotransmitter uptake from the synapse cleft into the cytoplasm needs an electrochemical gradient created by Na^+^-K^+^-ATPase present on the plasma membrane. Bilirubin inhibits Na^+^-K^+^-ATPase activity by disturbing the membrane structure (Tsakiris, 1993; Hoffman et al., 1996; Brito et al., 2004). Another pump for neurotransmitter uptake is the vesicular H^+^-ATPase, which provides a proton gradient for neurotransmitters to enter the vesicles. Roseth et al. (1998) reported that 100 μM of bilirubin did not affect the proton gradient across vesicle membranes, indicating that bilirubin does not affect vesicular H^+^-ATPase activity. Bilirubin has identical inhibitory effects on the vesicular uptake of catecholamines and glutamate, which are transferred by different transporters. Therefore, it is possible that the interaction of bilirubin with the transmembrane domains of these transporter proteins results in non-selective inhibition of these transporters (Roseth et al., 1998). In addition, bilirubin inhibits the phosphorylation of synapsin-I, a synaptic vesicle-associated protein that promotes neurotransmitter release upon phosphorylation (Hansen et al., 1988a). This may be another potential mechanism by which bilirubin affects neurotransmitter metabolism.

#### 3.1.1. Glutamate

L-glutamate is the primary excitatory neurotransmitter in the mammalian CNS. The two major classes of ionotropic glutamate receptors are alpha-amino-3-hydroxy-5-methyl-4-isoxazolepropionic (AMPA) and NMDA receptors. Glutamate plays a vital role in the development and maturation of the nervous system, including the generation, migration, and survival of neurons, neurite outgrowth, synaptogenesis, and synaptic plasticity (Hansen et al., 2021). However, a high level of extracellular glutamate in the synaptic cleft can cause glutamate receptor hyperactivation and trigger excessive Ca^2+^ entry into the neurons. This leads to a cascade of apoptotic and necrotic processes known as glutamate excitotoxicity (Mahmoud et al., 2019). Astrocytes take up the majority of glutamate from the synaptic cleft and convert it to glutamine, which is recycled and reconverted into glutamate (Andersen et al., 2021). Bilirubin increases glutamate release in both neurons and astrocytes, particularly in immature cells (Falcao et al., 2006). It also inhibits glutamate uptake by astrocytes in a pH- and concentration-dependent manner (Silva et al., 1999). Increased glutamate levels, detected using ^1^H-MR spectroscopy, have also been reported in kernicterus infants (Oakden et al., 2005) and *in vivo* animal experiments (Hu et al., 2014).

Previous studies have demonstrated that bilirubin-induced neurotoxicity increases the risk of NMDA receptor-triggered excitotoxic brain injury, while the NMDA receptor antagonist, MK-801 (dizocilpine), reduces bilirubin toxicity (Hoffman et al., 1996; McDonald et al., 1998; Grojean et al., 2000). A series of studies has suggested that AMPA receptors may also mediate bilirubin neurotoxicity. Myelination deficits induced by UCB in rat cerebellar slice cultures were partially rescued by AMPA inhibitors (Barateiro et al., 2014). Bilirubin-evoked action potential firing in the lateral superior olive (Li et al., 2011) and ventral cochlear nucleus (VCN) (Han et al., 2015) neurons was also inhibited by a combination of non-NMDA and NMDA receptor antagonists. As bilirubin increased the frequency rather than the amplitude of spontaneous and miniature excitatory postsynaptic currents, and did not affect glutamate-evoked currents, bilirubin-induced neuronal excitability may be mediated by increased presynaptic transmitter release, not by sensitizing postsynaptic glutamate receptors. This would be consistent with the conclusions of Warr et al. (2000), who found that free bilirubin (10 μM) failed to increase the current evoked by NMDA or AMPA in CA1 pyramidal cells in hippocampal slices and glutamate transporter currents in retinal glial cells. However, MK-801 treatment failed to protect the viability of hippocampal tissues (Shapiro et al., 2007; Dani et al., 2019), and provided no neuroprotection for auditory function, assessed using brainstem auditory-evoked potentials in Gunn rat pup models (Shapiro et al., 2007). The differences in these experiments, such as bilirubin concentration (with or without albumin in the medium), measurement index, and experimental models, may have contributed to the conflicting results.

#### 3.1.2. γ-Aminobutyric acid

GABAergic inhibitory neurons (interneurons) make up about 20% of the neuronal population in the mammalian CNS. These neurons control signal processing by secreting the neurotransmitter γ-aminobutyric acid (GABA), and balance neuronal excitatory activity and regulate plasticity (Hennequin et al., 2017). Bilirubin increased the frequency of inhibitory postsynaptic currents, both in the lateral superior olive and VCN, in a manner dependent on presynaptic intracellular Ca^2+^ (Shi et al., 2006; Li et al., 2010). This demonstrates that bilirubin can facilitate inhibitory GABAergic synaptic transmission by activating presynaptic protein kinase A (Li et al., 2010).

The postsynaptic function of GABA depends on intracellular chloride ([Cl^–^]i) levels, which vary between immature and mature neurons. In general, Na-K-2Cl cotransporter-1 (NKCC1; used for inward transport of Cl^–^) expression is high in immature neurons, while K-Cl cotransporter-2 (KCC2; mediates Cl^–^ efflux) expression is very low. KCC2 expression is upregulated with age, coupled with a decrease in NKCC1 activity. Therefore, GABA triggers Cl^–^ efflux and leads to neuronal depolarization in immature neurons, which are high in [Cl^–^]i, but mediates inhibition in mature neurons with low [Cl^–^]i levels (Ben-Ari et al., 2012). We also observed this shift in GABA function from excitatory to inhibitory in cochlear nucleus neurons (Song et al., 2012). Notably, in the immature VCN neurons (P2–6), bilirubin dramatically increased the spontaneous firing rate, and blocking GABA/glycine receptors greatly attenuated the bilirubin-induced hyperexcitability (Yin et al., 2016). This indicates that, in the early developmental stages, excitatory GABA/glycinergic transmission is involved in bilirubin-induced hyperexcitability.

#### 3.1.3. Catecholamines

Hansen et al. (1999) reported that bilirubin directly inhibits the release and vesicular storage of norepinephrine. Ca^2+^-induced exocytotic release of norepinephrine was inhibited at low levels of bilirubin (half-maximal effect at approximately 25 μM), while higher levels of bilirubin (100–320 μM) induced norepinephrine leakage from vesicles. A dose-dependent inhibitory effect of bilirubin on tyrosine (dopamine precursor) uptake has also been reported; the extent of the inhibition ranges from 86% at 70 μM to 50% at 140 μM (Amato et al., 1994). Bilirubin inhibited both cAMP-dependent protein kinase activity and cAMP-stimulated dopamine synthesis, but did not affect the basal dopamine synthesis rate (Brann et al., 1985). The uptake and vesicular storage of dopamine were also inhibited by bilirubin (Ochoa et al., 1993; Roseth et al., 1998). A recent series of clinical studies has demonstrated elevated serum bilirubin levels in Parkinson’s disease (PD) patients (Moccia et al., 2015; Lee et al., 2019; Macias-Garcia et al., 2019; Jin et al., 2020), and total serum bilirubin had a positive correlation with presynaptic dopamine transporter uptake (Lee et al., 2019). Dopaminergic neurons are particularly susceptible to oxidative stress because of the numerous oxidants produced during dopamine metabolism, which is thought to be an important pathogenic mechanism for PD (Dionisio et al., 2021). Bilirubin is a powerful endogenous antioxidant and is increased as an adaptive response in some diseases (Nocentini et al., 2022). Therefore, it has been suggested that increased bilirubin may be a protective factor against oxidative stress in PD (Moccia et al., 2015; Lee et al., 2019; Jayanti et al., 2021).

### 3.2. Effect of bilirubin on synaptic plasticity

The neural circuits are malleable computational structures rather than immutable, permanent networks. This is related to synaptic plasticity, the activity-dependent changes in the strength of neuronal connections. In rat hippocampus slides, bilirubin treatment (at least 100 μM, bovine serum albumin: bilirubin ratio of 1:8) reduced the slope of field excitatory postsynaptic potentials (fEPSPs) and shifted the presynaptic fiber volley/fEPSP curve to the right. This indicates that bilirubin inhibits synaptic activation and increases postsynaptic excitability (Hansen et al., 1988b). Chang et al. (2009) reported that long-term potentiation (LTP) and long-term depression (LTD) induction were impaired by UCB (1–10 μM for 24–48 h) in a time- and concentration-dependent manner (Chang et al., 2009), possibly through calpain-mediated proteolytic cleavage of NMDA receptor subunits. *In vivo* studies have also demonstrated that hyperbilirubinemia can cause LTP inhibition (Zhang et al., 2003; Yang et al., 2020) and the impairment of input/output functions and paired-pulse reactions, which reflect the basal synaptic response and short-term synaptic plasticity (Yang et al., 2020).

A 30-year prospective follow-up study in Finland found that 45% of the neonatal hyperbilirubinemia group participants had childhood cognitive abnormalities, poorer academic performances, and a greater risk of unemployment in the adulthood (Hokkanen et al., 2014). Synaptic plasticity is the primary mechanism for information storage and learning, and is critical for the refinement of developing neural circuits (Feldman, 2020). Therefore, synaptic plasticity defects may act synergistically with the reduced dendritic arborization and myelin disruptions in the hippocampus to induce memory deficit and impair cognition and learning.

## 4. Bilirubin and ion-channel activity

A variety of voltage-gated ion channels on neuronal membranes confer intrinsic electrical properties to the neurons and promote neuronal excitability. These channels are localized in the somatodendritic and axonal domains, serving specific neuronal functions (Child and Benarroch, 2014). Several studies have demonstrated that bilirubin can directly affect various ion-channel activities (Figure 2).

### 4.1. Calcium channels

Increased intracellular Ca^2+^ ([Ca^2+^]i) plays an important role in triggering downstream events of bilirubin-induced neurotoxicity (Watchko, 2006). Existing research indicates that bilirubin-induced [Ca^2+^]i overload involves interference with Ca^2+^ influx through voltage-gated calcium channels (VGCCs) and the release of intracellular calcium stores (Rauti et al., 2020). In response to action potentials and subthreshold depolarizing signals, VGCCs function as key transducers for the conversion of membrane potential to [Ca^2+^]i transients and initiate signaling cascades that regulate several physiological events, including transmitter release and synaptic plasticity (Catterall, 2011). We investigated the effect of different concentrations (1–6 μM) of bilirubin on VGCCs in bushy cells of the VCN in postnatal rat pups (P4–17), and found that 3 and 6 μM of bilirubin enhanced VGCC currents, mediated by high voltage-activated P/Q-type calcium currents in Ca^2+^- and calmodulin-dependent mechanisms (Liang et al., 2017a). Furthermore, as P/Q-type calcium channels are more abundant in neonatal neurons than in later stages, this type-specific effect may contribute to the early neuronal vulnerability to bilirubin. Albanna et al. perfused isolated murine retinae from wild-type (WT) and Ca_v_2.3-deficient mice with UCB and recorded the electroretinograms. They found that UCB significantly decreased the b-wave amplitude, which reflects the response of bipolar cells, in WT retinae, but not in the Ca_v_2.3-deficient group. This suggests an effect of bilirubin on neuronal signaling mediated by selective modulation of Ca_v_2.3/R-type channels (Albanna et al., 2018, 2019). Nicotinamide adenine dinucleotide (NAD+) regulates several cellular processes, including calcium homeostasis, mitochondrial functions, and energy metabolism. In our previous study, we found that NAD+ attenuates bilirubin-induced hyperexcitation of the VCN neurons by inhibiting VGCC currents and glutamate release, and decreasing postsynaptic intrinsic excitability (Liang et al., 2017b,^2022^).

### 4.2. Sodium channels

Bilirubin-induced increase in the intrinsic neuronal excitability was also associated with enhanced voltage-gated sodium channel (VGSC) currents in neonatal neurons in the rat medial vestibular nucleus (Shi et al., 2019). Bilirubin enhanced VGSC currents by facilitating the recruitment of Na_V_1.1 channels from the cytosolic pool to the cytoplasmic membrane in a Ca^2+^-dependent manner. Moreover, bilirubin-induced VGSC upregulation exacerbated cell death, which was significantly attenuated by the VGSC blocker, lidocaine. This indicates that VGSCs may be a potential target for bilirubin neurotoxicity treatment.

### 4.3. Acid-sensing ion channels

Bilirubin toxicity is often aggravated by acidosis (Dennery et al., 2001; Brito et al., 2006). We found that the concentration of the cell death marker, lactate dehydrogenase, in the cerebrospinal fluid of infants with both hyperbilirubinemia and acidosis was much higher than in those with one of these conditions, which indicates the synergistic neurotoxicity of acidosis and bilirubin (Lai et al., 2020). Previous studies have reported that respiratory acidosis alters the blood-brain barrier permeability and promotes bilirubin accumulation in the brain tissues in animal models (Bratlid et al., 1984; Wennberg, 2000). This may contribute to the aggravated bilirubin neurotoxicity in cases with acidosis. We recently identified a molecular mechanism that may further explain this phenomenon (Lai et al., 2020). In an acidic environment, bilirubin enhanced acid-sensing ion channel (ASIC) currents via a cascade of [Ca^2+^]i-CaMKII-ASIC1a signaling in the medial vestibular nucleus neurons of neonatal mice (Lai et al., 2020). Bilirubin and protons synergistically contribute to neuronal overexcitation, [Ca^2+^]i overload, and the resultant neurotoxicity (Lai et al., 2020). Furthermore, concurrent hyperbilirubinemia and acidosis during the early neonatal period can increase the severity of long-term sensory and cognitive dysfunction compared to either condition alone in mouse models (Lai et al., 2020). This suggests that ASIC1a may be a potential target for the treatment of concurrent hyperbilirubinemia and acidosis in infants.

## 5. Discussion and conclusion

Bilirubin affects various stages of neural circuit development and directly alters neuronal electrical activity. Bilirubin-induced NSC damage may disrupt the proliferation/differentiation balance and alter the numbers and types of neuronal cells, which is an important cause of several neurodevelopmental and neuropsychiatric disorders (Sacco et al., 2018). Bilirubin-induced impairment of neural cell maturation, including the inhibition of axonal and dendritic arborization, reduced synapse formation, and myelin damage, disrupts neuronal connections and the circuit structure. The dysfunction of synaptic transmission and ion channels disrupts the integration of excitatory and inhibitory signals and increases neuron excitability, which may affect information transduction and neural network processing.

The structural and functional insults by bilirubin lead to different outcomes in different brain nuclei. Bilirubin deposition in the globus pallidus, which is part of cortico-basal ganglia circuits involved in many brain functions such as the acquisition of motor skills and perceptual-motor learning, stimulus-response learning, and reward-based learning, resulting in cognitive and behavioral deficits, including the impulse- and stimulus-bound behaviors seen in ADHD and language impairment (Koziol et al., 2013; Amin et al., 2019). Impaired synaptic transmission and neuronal excitotoxicity in the auditory brainstem nuclei may cause hearing loss and affect language development. Impaired synaptic plasticity and neuronal death in the hippocampus may cause memory and learning disabilities in patients with kernicterus spectrum disorders. Cerebellar injury, with reduced numbers of Purkinje and granule cells and destruction of dendrites, may disrupt the multisensory feedback loop between the cerebellum and cortex, which may explain the clinical characteristics of ASDs (Amin et al., 2011). However, the relationship between bilirubin-induced neuropathological changes and long-term clinical neurological outcomes has not been fully elucidated and needs further investigation.

In recent years, studies have explored the physiological roles of bilirubin, including as an antioxidant (Stocker et al., 1987; Sedlak et al., 2009; Vasavda et al., 2019), anti-inflammatory, immunomodulator (Liu et al., 2008; Jayanti et al., 2020; Tran et al., 2020), and inhibitor of protein phosphorylation (Hansen et al., 1988a,^1996^, ^1997^). It is widely accepted that physiological concentrations of bilirubin may be protective for the CNS (Gazzin et al., 2016; Creeden et al., 2021). *In vitro* hippocampal cultures and bilirubin-deficient *Blvra^–/–^* mouse experiments suggest that bilirubin protects the neurons from oxidative stress (Doré and Snyder, 1999; Vasavda et al., 2019). Clinical studies have shown that total serum bilirubin levels are negatively associated with the prevalence of stroke (Thakkar et al., 2019; Choi et al., 2020) and the disease duration of multiple sclerosis (Ljubisavljevic et al., 2013). Total serum bilirubin concentrations are lower in infants who develop neonatal hypoxic-ischemic encephalopathy (Dani et al., 2018; Haga et al., 2022). These studies suggest the potential role of bilirubin in maintaining healthy brain function. To understand the effects of bilirubin on CNS, discriminative studies simulating both physiological and pathological conditions are required.

There are several other interesting research areas for the physiological role of bilirubin in the CNS. Electrical activity integrates with gene expression to regulate neuronal development at nearly all stages of development (Spitzer, 2006). It may be worth investigating how bilirubin regulates electrical activity under physiological conditions and its physiological significance. In addition, several studies have shown that bilirubin may affect gut microbiome diversity (Nobles et al., 2013; Lee et al., 2020; Vitek and Tiribelli, 2020). Gut microbiome regulates the development of various brain functions, including stress response, social behavior, motor control, and anxiety behaviors (Sudo et al., 2004; Diaz Heijtz et al., 2011; Desbonnet et al., 2014). Given that the neonatal period is a critical window of time for gut microbial colonization, bilirubin elevation, and rapid neurodevelopment, the association among them requires further exploration.

Bilirubin can act like a hormone by activating several nuclear and cytoplasmic receptors (Vitek, 2020). Bilirubin binds with and activates the Mas-related G-protein-coupled receptor member X4 (MRGPRX4) to elicit pruritus, and may also activate the aryl hydrocarbon receptor (AhR), thus increasing *Cyp1a1* expression in hepatocytes (Phelan et al., 1998; Meixiong et al., 2019). Bilirubin can directly and selectively bind to peroxisome proliferator-activated receptor-alpha (PPARα) at physiological concentrations to induce transcriptome responses (Gordon et al., 2019, 2020). PPARα regulates several important processes in the neural circuits, including neural cell proliferation and differentiation, neurotransmission, and synaptic plasticity. It has previously been investigated as a novel therapeutic target for neurodegenerative and neuropsychiatric diseases (Fidaleo et al., 2014; D’Angelo et al., 2018; Lee et al., 2021). However, bilirubin receptors in the CNS remain unknown and it remains to be investigated whether their downstream signaling pathways are involved in bilirubin-induced neurological changes.

In conclusion, pathologically high bilirubin concentrations cause neurological and neurodevelopmental damage through a variety of mechanisms. It is difficult to translate the research findings into clinical practice for the management of bilirubin encephalopathy. Further investigations of the mechanism and intervention targets for bilirubin neurotoxicity are needed. As an endogenous molecule with physiological elevation during the critical period of CNS development, an in-depth understanding of the physiological role of bilirubin in CNS development and function is required.

## Author contributions

SY and CL conceived and designed the study. CW and YJ wrote the manuscript and illustrated the figures. YC and YZ performed the literature search. All authors contributed significantly to the article and approved the current version for submission.
